# DNAzymes, Novel Therapeutic Agents in Cancer Therapy: A Review of Concepts to Applications

**DOI:** 10.1155/2021/9365081

**Published:** 2021-11-01

**Authors:** I. B. K. Thomas, K. A. P. Gaminda, C. D. Jayasinghe, D. T. Abeysinghe, R. Senthilnithy

**Affiliations:** ^1^Department of Biology, Faculty of Applied Sciences, South Eastern University of Sri Lanka, Sri Lanka; ^2^Department of Chemistry, Faculty of Natural Sciences, The Open University of Sri Lanka, Nugegoda, Sri Lanka; ^3^Department of Zoology, Faculty of Natural Sciences, The Open University of Sri Lanka, Nugegoda, Sri Lanka

## Abstract

The past few decades have witnessed a rapid evolution in cancer drug research which is aimed at developing active biological interventions to regulate cancer-specific molecular targets. Nucleic acid-based therapeutics, including ribozymes, antisense oligonucleotides, small interference RNA (siRNA), aptamer, and DNAzymes, have emerged as promising candidates regulating cancer-specific genes at either the transcriptional or posttranscriptional level. Gene-specific catalytic DNA molecules, or DNAzymes, have shown promise as a therapeutic intervention against cancer in various *in vitro* and *in vivo* models, expediting towards clinical applications. DNAzymes are single-stranded catalytic DNA that has not been observed in nature, and they are synthesized through *in vitro* selection processes from a large pool of random DNA libraries. The intrinsic properties of DNAzymes like small molecular weight, higher stability, excellent programmability, diversity, and low cost have brought them to the forefront of the nucleic acid-based therapeutic arsenal available for cancers. In recent years, considerable efforts have been undertaken to assess a variety of DNAzymes against different cancers. However, their therapeutic application is constrained by the low delivery efficiency, cellular uptake, and target detection within the tumour microenvironment. Thus, there is a pursuit to identify efficient delivery methods *in vivo* before the full potential of DNAzymes in cancer therapy is realized. In this light, a review of the recent advances in the use of DNAzymes against cancers in preclinical and clinical settings is valuable to understand its potential as effective cancer therapy. We have thus sought to firstly provide a brief overview of construction and recent improvements in the design of DNAzymes. Secondly, this review stipulates the efficacy, safety, and tolerability of DNAzymes developed against major hallmarks of cancers tested in preclinical and clinical settings. Lastly, the recent advances in DNAzyme delivery systems along with the challenges and prospects for the clinical application of DNAzymes as cancer therapy are also discussed.

## 1. Introduction

Completion of the Human Genome Project has expanded our understanding of the genetic root of many incurable diseases such as cancer, Alzheimer's, Parkinson's, asthma, and rheumatoid arthritis [1]. A new stride in genomic medicine has concocted novel therapeutic options that can modulate the disease outcomes by regulating the disease-specific genes [2]. Recently, nucleic acids, including deoxyribonucleic acid (DNA) and ribonucleic acid (RNA), have emerged as stand-alone therapeutics that go beyond mere storage and transmission of genetic information [3]. Fundamentally, nucleic acid therapy inhibits either DNA or RNA expression and subsequently halts the production of an abnormal protein associated with a disease while other proteins are unaffected [3]. Further, both DNA and RNA exert catalytic properties that can perform specific chemical reactions, with efficacy comparable to that of protein enzymes [4].

Currently, DNA-based therapeutics is at the forefront of genomic medicine due to their specificity in recognizing molecular targets and pathways [3]. DNA-based therapeutics includes plasmids, oligonucleotides for antisense and antigene applications, DNA aptamers, and DNAzymes or deoxyribozymes [5]. Among DNAzymes, they impart tremendous potential in gene suppression [5]. Unlike ribozymes, DNAzymes have not been observed in nature, and all existing molecules result through *in vitro* selection processes from a random DNA library containing about 10^15^ DNA sequences [6]. DNAzymes catalyze an array of chemical reactions, including RNA cleavage, oxidative or hydrolytic DNA cleavage, DNA/RNA ligation, and DNA phosphorylation [6]. The extensively studied RNA-cleaving DNAzymes catalyze the cleavage of a single RNA linkage embedded within a DNA strand [6].

Following the isolation of DNAzymes in 1994, their usage has become ubiquitous in biomedical applications [7]. Their applications in cancer diagnostics and therapeutics are enormous. Cancer possesses a multifactorial etiology where hereditary and acquired defects singly or synergistically cause cellular transformations [8]. Hence, recent years witness a paradigm shift of anticancer drug development from conventional broad-spectrum cytotoxic compounds to molecular interventions that selectively act on specific targets. In the light, DNAzymes present an effective alternative to traditional chemotherapy owing to their specific catalytic activity [9]. Wu et al. [10] were the first to report the potential of DNAzymes as an anticancer agent *in vitro*. Since then, several DNAzymes have been synthesized, targeting various genes leading to cancer onset and progression [9]. Mainly, RNA-cleaving DNAzymes have been broadly investigated due to their potential to downregulate the target protein by cleaving corresponding mRNA [11]. Although DNAzymes possess high stability and selectivity than ribozymes, the therapeutic applications are limited by the inept delivery to intracellular targets [4]. Hence, critical analysis of their applications in cancer research would provide insight into their effectiveness and prospects as cancer therapy.

This review intends to provide critical analysis of available literature on applications of DNAzymes as an emerging anticancer therapy. Initially, this review will give a brief overview of the structure and synthesis of DNAzymes. Secondly, this review discusses the efficacy, safety, and tolerability of DNAzymes developed against different cancer types. The different DNAzymes developed against the specific molecular target in major hallmarks of cancer such as metastasis, angiogenesis, and apoptosis are discussed separately in this review. Further, the applicability of DNAzymes against cancer-specific alleles and oncogenic viruses has been addressed in this review.

Moreover, we summarize DNAzymes that have currently being evaluated in clinical trials and have obtained regulatory approval. Although DNAzymes have revolutionized the anticancer therapeutic arsenal, they are limited by inefficient delivery to the cancer cells. Consequently, attempts taken so far to enhance cellular delivery of DNAzyme therapeutics accompanied by the apparent challenges involved will be addressed briefly. It is anticipated that this comprehensive review would enlighten the reader about the efficacy of DNAzymes as novel nucleic acid-based therapy against cancer.

## 2. DNAzymes: Brief Overview

In 1994, Breaker and Joyce identified the catalytic property of DNA and isolated the first DNAzymes via an *in vitro* selection system [7]. The *in vitro* selection system was based on the hydrolytic cleavage of a phosphodiester and nested PCR [12]. A pool of 10^15^ ssDNA molecules containing a 5′ biotin moiety, followed by a 50-random-deoxyribonucleotide domain flanked by a fixed sequence, was established. Then, these molecules were exposed to a streptavidin affinity matrix and washed with buffer to remove the unbound. Next, the same buffer containing a certain cation passed through the matrix to cause cation-dependent cleavage of phosphodiester, and catalytic DNAs were released from the mixture. These DNAs were collected, reintroduced to the 5′ biotin and target phosphodiester, amplified by nested PCR, and finally subjected to several selection rounds [12].

**Figure 1:**
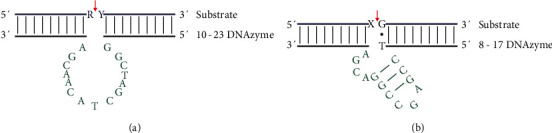
Structures of “10-23” (a) and “8-17” (b) DNAzymes. A, C, T, and G represent deoxyribonucleotides. R (A or G), Y (U or C), and X represent ribonucleotides. The red arrows indicate the cleavage site in the substrate strand.

DNAzymes are characterized by two domains: catalytic and substrate binding domains. However, their sequences can be varying. There are two main types: “10-23” and “8-17” DNAzymes (Figure 1) isolated from the *in vitro* selection system [12]. The “10-23” DNAzyme has been obtained from the 23^rd^ clone after 10 rounds of amplification, while “8-17” DNAzyme has been obtained from the 17^th^ clone after 8 rounds of amplification [12]. The catalytic core of “8-17” DNAzyme consists of 13 nt, having a short internal stem loop connected to an unpaired region of 4 nt. The loop contains a fixed sequence of 5′-AGC-3′. The sequence of an unpaired region represents 5′-WCGR-3′ or 5′-WCGAA-3′ (W = A/T, R = A/G) [12]. In the “10-23” DNAzyme, the catalytic core is composed of 15 nt, while the 8^th^ of which was usually T, C, or A and T often provides the highest activity [12].

**Figure 2:**
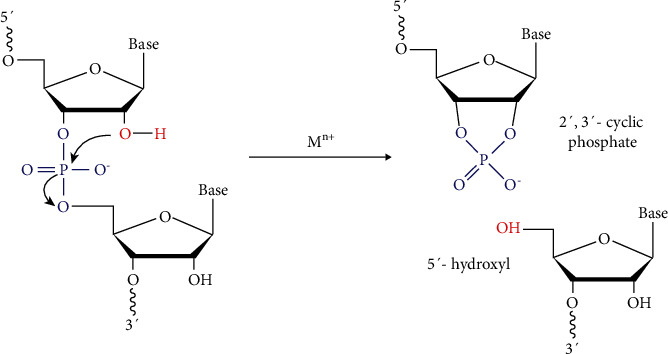
A generalized diagram showing the RNA cleavage catalyzed by a RNA cleaving DNAzyme forming a 2′,3′-cyclic phosphate and 5′-hydroxyl terminus, respectively.

The catalytic core binds to and cleaves its target RNA through a deesterification reaction. Two arms flank the core, each composed of a substrate recognition domain of 7-9 nt facilitating the binding to RNA substrate via the Watson-Crick hybridization. Ultimately, the substrate strand splits at the cleavage site giving rise to 5′ and 3′ products which comprise a 2′,3′-cyclic phosphate and 5′-hydroxyl terminus, respectively (Figure 2) [13]. The “10-23” DNAzyme can cleave almost any phosphodiester bond between an unpaired purine and paired pyrimidine [14]. Most DNAzymes are assisted by specific metal ions such as Mg^2+^, Pb^2+^, Mn^2+^, Cu^+2^, and Na^+^ that function as cofactors contributing to achieving a satisfactory reaction rate [11]. In addition, these metal cations promote the formation of DNAzyme structure as DNA is a negatively charged polyelectrolyte whose folding is strongly dependent on electrostatic [15]. Collectively, the overall reaction mechanism of metal cation-dependent RNA cleaving DNAzymes can be considered an evidence to support metal-assisted deprotonation of the 2′-hydroxyl located adjacent to the cleavage site [13]. This produces a nucleophilic 2′-oxyanion that attacks the adjacent phosphorus giving rise to intended cleavage products. The metal cation may participate in the chemical reaction either as a metal hydroxide that function as a general base to assist deprotonation of the 2′- hydroxyl or as a Lewis acid that coordinates directly to 2′-hydroxyl enhancing its acidity [13].

The catalytic property of DNAzyme is particular; mismatch sequences in the binding arms or point mutations in the catalytic core make the DNAzyme catalytically inactive and can be used as control molecules when accessing the biological specificity of DNAzymes [9]. A variety of structural modifications are incorporated in order to enhance the stability and potency of DNAzymes. These include 3′-3′ inverted nucleotide at the 3′ end, phosphorothioate linkages, and locked nucleic acids. Incorporating inverted thymidine at the 3′ terminus of the DNAzyme leading to a 3′-3′ linkage increases the stability and resistance against 3′-exonuclease in human serum [16]. Phosphorothioate linkages in DNAzymes are characterized by the presence of a sulfur atom in one of the nonbridging phosphate oxygen atoms in the cleavage site [17]. These enhance the stability of DNAzymes by providing more resistance to endogenous nucleases. Phosphorothioate modifications are hardly applied in the field of DNAzymes as they are well known to cause nonspecific protein binding leading to toxicity [18]. Locked nucleic acids contain a 2′-O, 4-C methylene bridge that locks sugar rings in a C3′-endo confirmation. Incorporating locked nucleic acids into DNAzymes increases binding affinity, stability towards 3′-exonucleolytic degradation, and solubility and is considered an attractive strategy [9]. A concise summary of the advantages and disadvantages of these modifications is given in Table 1.

Compared to other enzymes, DNAzyme shows some inherent advantages such as structural stability, accurate target cleavage without any immune response, and most negligible cytotoxicity. DNAzymes exert high specificity and can be easily modified or functionalized. Further, the synthesis of DNAzymes is cost-effective compared to other nucleic acid-based therapies [11].

## 3. Application of DNAzymes in Cancer Research: Experimental Evidence

Cancer is one of the leading life-threatening diseases in the world (https://www.who.int/news-room/fact-sheets/detail/cancer). The development of efficient therapeutic strategies for cancer treatment is a fundamental aspect of cancer research. Treatment options at present, such as chemotherapy and radiation therapy, are not universally effective for all cancers [19]. Hence, there is a pertinent requirement to investigate novel therapeutic options.

Cancer is specified by the abnormal proliferation of any of the different kinds of cells in the body accompanied by metastasis, thereby giving rise to several distinct types of cancer that vary in their behaviour and response to treatment [20]. It involves complex genetic and epigenetic alterations that include mutations, chromosomal translocation or deletion, and downregulation or overexpression of tumor suppressor genes and protooncogenes [21]. These mechanisms contribute to activating genes that promote dysregulated cell cycling and/or inactivate apoptotic pathways [22] and drive the progressive transformation of normal human cells into highly malignant derivatives [10].

**Figure 3:**
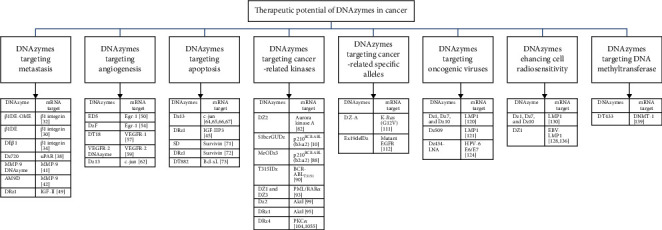
Schematic diagram showing the therapeutic potential of DNAzymes targeting various attributes of cancer.

DNAzymes have exhibited great potential in downregulating cancer-associated genes. The anticancer DNAzymes make use of the hallmarks of cancer that include self-sufficiency in growth signals, insensitivity to growth inhibitory signals, tissue invasion, metastasis, sustained angiogenesis, evasion of apoptosis, and limitless replicative potential [23] to suppress tumor growth.  Figure 3 comprises a schematic diagram showing different DNAzymes that have been explored so far to target various attributes of cancer whereas Table 2 summarizes the respective sequences of DNAzymes.

The subsequent sections will discuss the successful applications of DNAzymes in addressing the aforementioned cancer hallmarks. Tables 3 and 4 present an overview of *in vitro* and *in vivo* applications of DNAzymes in cancer treatment, respectively.  Table 5 summarizes the clinically tested DNAzymes in cancer treatment so far.

### 3.1. DNAzymes Targeting Metastasis

The strategy of targeting metastasis has received increasing attention in cancer biology. Cancer metastasis is the process by which cancer cells spread to other parts of the body from the original tumor site [24]. It entails a series of steps, generally known as a metastasis cascade, which contributes to cancer severity. To complete the metastasis cascade, cancer cells must detach from the primary tumor, intravasate into the circulatory and lymphatic system, evade immune attack, extravasate at distant capillary beds, and invade and proliferate in distant organs [25]. The regulation of cell-cell and cell-matrix adhesions is crucial during metastasis.

Among the number of proteins involved in cancer metastasis, integrins play a major role and have been identified as an effective pharmacological target. Integrins are transmembrane cell adhesion receptors of 18*α* and 8*β* subunits that combine to form about 24 different heterodimeric receptors [26]. *β*1 is known to play a critical role in the migration, proliferation, metastasis, and angiogenesis of cancer [27].

Wiktorska et al. [28] formulated *β*1 integrin-*β*1DE-OME DNAzyme and transfected it into several colon carcinoma cell lines (CX1.1, HT29, LOVO, and LS180) and a prostate cancer cell line (PC-3). It was sobered that *β*1DE-OME treatment reduced the invasiveness of tested cells. Further, PC-3 was more sensitive to *β*1DE-OME compared to other cancer types [28]. Moreover, the inhibitory activity of *β*1 integrin-*β*1DE was investigated *in vivo* using Matrigel plugs (BALB/c mice) and nude mice (BALB/cA nude (nu-/-)-B6.Cg-*Foxn1*^nu^) with solid carcinoma developed using PC-3 cells and CX1.1 cells [29]. Administration of *β*1DE effectively inhibited the neovascularization stimulated by bFGF added to Matrigel plugs. Further, intratumoral injection of *β*1DE produced a noticeable tumor size reduction in the CX1.1 and PC-3 xenografts within the 3 weeks of experiment duration [29]. In a parallel experiment, the efficacy of DNAzyme (DE*β*1) in inhibiting the expression of *β*1 integrin was compared with siRNA (siRNA*β*1) using HT29 and PC-3 cancer cells both *in vitro* and *in vivo* [30]. Experimental results revealed that siRNA*β*1 was slightly more efficient than DE*β*1 in the *in vitro* assay. However, DE*β*1 exhibited higher efficiency in blocking the tumor growth *in vivo* due to its resistance for degradation in extra- and intracellular compartments when compared to that of siRNA [30].

A urokinase plasminogen activator system (uPA) is a serine protease group involved in multiple steps in cancer progression [31]. Elevated uPAR expression levels were observed in many types of cancer, including non-small-cell lung cancer (NSCLC) and colorectal cancer (CLC), leading to poor prognosis, early invasion, and metastasis [32]. uPA directs the catalysis of the formation of plasmin from plasminogen. Plasmin brings about the degradation of the extracellular matrix (ECM) and basement membrane, thereby facilitating the penetration of tumor cells to the ECM and basement membrane to metastasize [33].

de Bock et al. [34] designed three DNAzymes, Dz372, Dz483, and Dz720, against uPAR mRNA. Among them, two DNAzymes, Dz483 and Dz720, cleaved the target uPAR transcript with high efficacy and specificity *in vitro*. The ability of Dz720 and Dz483 to suppress uPAR mRNA expression was tested in a human osteosarcoma cell line (Saos-2). It was observed that Dz720 could inhibit the uPAR expression at both mRNA and protein levels. Saos-2 cells treated with Dz720 brought about an inhibition in cell invasion due mainly to decreased invasion and partly reduced cell proliferation [34]. This system has shown a synergistic effect rather than only an additive effect [34].

Matrix metalloproteinases (MMPs) are members of the metzincin protease superfamily of zinc-endopeptidases which are involved in many biological processes such as degradation of ECM [35]. Among the 28 MMPs identified, MMP-9 is considered a potential biomarker whose overexpression can be seen in a wide array of tumors, including NSCLC and colorectal, cervical, and breast cancer [35]. MMP-9 degrades type IV collagen and contributes to tumor progression through invasion, metastasis, growth, and angiogenesis [36]. To determine the potential of DNAzymes as antimetastasis agents in the treatment of NSCLC by targeting the MMP-9 gene, Yang et al. [37] synthesized MMP-9 DNAzyme and transfected it into an NSCLC cell line (A549). The DNAzyme downregulated the expression of MMP-9 at both mRNA and protein levels and inhibited cell proliferation, adhesion, migration, and invasive capacity *in vitro*. MMP-9-AM9D DNAzyme has also been tested to determine its efficacy as an antitumor agent for breast cancer therapy both *in vitro* and *in vivo*. Upon transfection into a human breast cancer cell line (MDA-MB-231), the DNAzyme downregulated the MMP-9 mRNA expression, concomitantly inhibiting the invasive behavior of MDA-MB-231 cells *in vitro*. MMTV-PyMT transgenic breast cancer mouse model was used to figure out the capacity of AM9D as an inhibitor of MMP-9 *in vivo* [38]. Broad-spectrum MMP inhibitor, Galardin/GM6001, reduced primary mammary tumor growth and lung metastasis in the MMTV-PyMT model. Compared with the broad-spectrum inhibitor, GM6001 [39], AM9D treatment has downregulated the MMP-9 without affecting the expression of other members of the MMP family.

Derangement of the insulin-like growth factor (IGF) signaling pathway, predominantly at the level of IGF-II availability, is implicated in many types of cancers [40]. IGF-II is a fetal growth protein and an essential proangiogenic factor [41]. The human IGF-II gene contains four promoters (P1-P4) [42], out of which P1 is active and P2-P4 are decreased or completely deactivated in the adult liver [43]. Reactivation of the main active promoters in fetal development, such as P3, contributes to increased levels of IGF-II in hepatocellular carcinoma (HCC) cells [41]. IGF-II directly promotes angiogenesis and indirectly promotes the formation of liver cancer blood vessels, thereby contributing to tumor progression. A variety of liver cancer cell lines secrete IGF-II through an autocrine or paracrine mechanism [44]. In their study, Zhang et al. [45] evaluated DRz1 to determine its effect on inhibiting the invasion, motility, and migration of an HCC cell line (SMMC-7721). Treatment with DRz1 downregulated the IGF-II expression at both mRNA and protein levels and inhibited the invasion, motility, and migration of SMMC-7721 cells. Also, DRz1 reduced the adhesion of tested cells to ECM proteins such as fibronectin and laminin and human fibroblast cells. Further, DRz1 downregulated the expression of vascular endothelial growth factor (VEGF) and MMP-9 at both mRNA and protein levels, thereby inhibiting the invasion and motility of SMMC-7721 cells [45].

### 3.2. DNAzymes Targeting Angiogenesis

Angiogenesis is the process by which new blood vessels are formed from the preexisting vasculature. Cancer cells, albeit malignant, depend on blood vasculature for oxygen, nutrients, and waste removal similar to normal cells, and therefore, angiogenesis is imperative in tumor progression [46]. Angiogenesis is under the regulation of both activator and inhibitor molecules, and the expression levels of these angiogenic factors determine the aggression of tumor cells [47].

Early growth response-1 (Egr-1) is a member of the family of Cys_2_-His_2_–type zinc finger transcription factors containing a highly conserved DNA-binding domain that binds to GC-rich recognition motifs [48]. Egr-1 regulates cell growth and differentiation upon the activation by extracellular agonists such as growth factors and cytokines and environmental stresses such as radiation, vascular injury, fluid shear stress, and hypoxia [46]. It is expressed in several different kinds of breast carcinoma cells, for instance, MCF-7 and MDA-MB-231 [49]. It is associated with transformed growth, multidrug resistance-1 (MDR-1) gene transcription, and antiestrogen responsiveness in MCF-7 cells [50]. Assembling both *in vitro* and *in vivo* analyses, Fahmy et al. [46] identified the mandatory role played by Egr-1 in proliferation, migration, and neovascularization of microvascular endothelial cells. Based on that, Egr-1-ED5 DNAzyme was exploited in deciding its capacity against tumor growth and angiogenesis *in vivo*. Administration of ED5 into athymic nude mice resulted in a profound reduction in tumor growth without affecting body weight, wound healing, hemostasis, and reproduction. It was identified that ED5 inhibited the MCF-7 tumor growth by blocking host angiogenesis. Both Egr-1 and fibroblast growth factor-2 (FGF-2) expression levels were substantially low in MCF-7 tumors treated with ED5. Administration of FGF-2 produced a robust tumor regrowth suggesting the reversible and FGF-2-mediated inhibition of endothelial growth and tumor angiogenesis by ED5 [46].

Egr-1-DzF is another DNAzyme which produced a profound sequence-specific inhibition of solid breast carcinoma growth *in vivo*. The ability of Egr-1 DNAzymes to attenuate angiogenesis suggests the emerging feasibility of small-molecule gene-targeting agents as cancer therapeutics [50].

VEGF is identified as an endothelial cell-specific mitogen produced by many cell types, including tumor cells, macrophages, platelets, keratinocytes, and renal mesangial [51]. VEGF-A is one of the five members of the mammalian VEGF family regulating angiogenesis by interacting with two major tyrosine kinase receptors, namely, VEGFR-1 (FLT-1) and VEGFR-2 (KDR/ FLK-1) [52]. In relation to the surrounding normal tissue vasculature, VEGFR-1 and VEGFR-2 have upregulated tumor-associated endothelial cells (ECs) in various tumors [53]. VEGFR-2 induces vascular permeability, proliferation, differentiation, survival, and migration of ECs and drives VEGF-mediated angiogenesis [54]. The VEGF/VEGFR signaling pathway is usually upregulated in many types of cancers, and therefore, therapeutic paradigms targeting VEGF/VEGFR are of prime importance.

Shen et al. [53] conducted a comprehensive study to determine the potentiality of DNAzymes targeting VEGFR-1 both *in vitro* and *in vivo* as cancer therapeutics. Out of 11 DNAzymes developed, DT18 was found to incur the most potent antiangiogenic activity *in vitro.* DT18 inhibited the growth of B16 melanoma tumors without affecting cell proliferation *in vivo.* Profound suppression of the nasopharyngeal carcinoma (NPC) tumor growth along with the VEGFR-1 expression further confirmed the antitumor efficacy of DT18. The downregulation of VEGFR-1 expression exerted a change in tumor vasculature and vessel permeability. Further, the administration of DT18 to healthy mice demonstrates the absence of any toxicity revealing its potentiality for cancer treatment, such as NPC.

Moreover, Zhang et al. [55] designed a VEGFR-2-DNAzyme and screened against human breast cancer cells (MDA-MB-435) as a possible antiangiogenic agent *in vivo*. Administration of the DNAzyme into nude mice reduced the cell proliferation and induced apoptosis leading to overall tumor growth reduction. A pronounced decrease in blood vessel density with large areas of peripheral cell death in DNAzyme-treated tumor confirmed the antiangiogenic capability of the DNAzyme [55].

The AP-1 belonging to the family of basic leucine zipper (bZIP) transcription factors controls vital cellular processes such as differentiation, migration, proliferation, and apoptosis [56]. C-jun is a significant component of the AP-1 complex and is dysregulated in many types of cancers such as colorectal, adenocarcinoma, lung, and breast cancer tumors [57]. Moreover, elevated levels of c-jun have been shown to induce an invasive cancer phenotype in MCF-7 cells and impose drug resistance in human leukemia cells [56]. As a consequence, c-jun knockdown has become an ideal aspect of perturbing many cancers. The c-jun-Dz13 DNAzyme was capable of inhibiting c-jun protein expression and subsequent c-jun DNA-binding activity. It also attenuated the proliferation, migration, invasion, and tubule formation and blocked the expression and proteolytic activity of MMP-2 in human microvascular endothelial cell (HMEC-1) *in vitro*.

Moreover, it was capable of inhibiting VEGF165-induced neovascularization *in vivo*. These antiangiogenic properties of Dz13 were employed in alleviating the growth of solid melanomas *in vivo*. The administration of Dz13 retarded the solid B16 melanoma growth and inhibited the tumor vascular density, indicating the involvement of Dz13-mediated c-jun knockdown in inhibiting growth and angiogenesis of solid tumors [58].

### 3.3. DNAzymes Targeting Apoptosis

Apoptosis is the natural mechanism of programmed cell death. It is a regulated process that eliminates any unnecessary or unwanted cells. Apoptosis is carried out by a series of cysteine protease known as caspases that cleave hundreds of various target proteins [59].

It has been reported that the loss of caspase-2 results in an increased ability of cells to acquire a transformed phenotype and gain malignancy, suggesting that caspase-2 is a tumor suppressor protein [60]. Dass et al. [60] presented a strong caspase-2 activation as an off-target effect of Dz13 both *in vitro* and *in vivo.* Exposure to Dz13 resulted in apoptosis in human tumor cells used in the study. Cytochrome C release caused by Dz13 indicated the permeabilization of mitochondria and confirmed apoptotic cell death. Interestingly, Dz13-mediated cell death occurred even in the absence of piddosomal components such as a p53-induced protein with a death domain (PIDD), RIP-associated Ich-1/CED homologous protein with death domain (RAIDD), and DNA-dependent protein kinase catalytic subunit (DNA-PKcs), which are known to activate caspase-2 [60]. Dz13 has also exhibited anticancer activity via the caspase-10-mediated apoptotic cell death in an orthotopic model of liposarcoma (LS) [61].

Furthermore, Dz13 was screened against basal cell carcinoma (BCC) and squamous cell carcinoma (SCC) in a therapeutic setting with established tumors in both immunocompromised and immunocompetent mice [62]. Intratumoral administration of Dz13 produced a prominent tumor growth suppression in immunocompetent syngeneic mice, suggesting adaptive immune system involvement in DNAzyme-mediated tumor growth suppression. Dz13 has increased the percentage of CD4^+^ and CD8^+^ cells in SCC tumors of immunocompetent mice. Incorporating a G>C point mutation to the catalytic domain of the DNAzyme (Dz13.G>C) eliminated the tumor inhibition indicating the necessity of an intact catalytic domain to drive DNAzyme-mediated tumor inhibition. Exposure to Dz13 inhibited the expression of mitogenic markers such as proliferating cell nuclear antigen (PCNA) and cyclin-dependent kinase 4 (CDK4) and tumorigenic markers like MMP-2, MMP-9, VEGF-A, and FGF-2 in SCC tumors. Further, Dz13 treatment enhanced the expression of the CDK inhibitor p21^WAFI/Cip1^ and proapoptotic markers such as caspase-3, caspase-8, caspase-9, and p53 thereby increasing the apoptosis of SCC tumors [62]. Importantly, Dz13 was found to be safe and well-tolerated, and it did not impact more than 70 different biologically relevant assays, which led to the evaluation of Dz13 in first-in-human clinical trials as a potential therapeutic against skin cancer [62].

Accordingly, Cho et al. [63] conducted a first-in-class, first-in-human (DISCOVER) phase I trial to evaluate the safety and tolerability of Dz13 in patients with BCC. The intratumoral injection of Dz13 reduced the c-jun expression in BCC of all the test participants. As per data, a single administration of Dz13 affected the expression of a range of proteins associated with tumorigenesis, for instance, increased levels of caspase-3, caspase-8, caspase-9, and p53 and decreased levels of antiapoptosis mediators Bcl-2 and MMP-9. The major changes brought about by Dz13 in the population of immune and inflammatory cells such as CD3^+^, CD4^+^, CD8^+^, and CD1a^+^ dendritic cells suggested the involvement of inflammatory and adaptive immune response with DNAzyme-mediated apoptosis. Further, five out of nine test participants showed a reduction in histological tumor depth. Dz13, as suggested by the study, is safe and tolerable without detectable systemic exposure following a single intratumoral injection and possibly debulks the tumor prior to excision. This indicates the emergence of promising treatment options for c-jun-associated pathologies.

Dysregulation of IGF-II is associated with the attenuation of apoptosis and increased proliferation in HCC, leading to uncontrolled tumor growth and chemoresistance [41]. Properties of HCC that arise due to its heterogeneity such as rapid development, early metastasis, and difficulty in establishing prognosis and survival time led Zhang et al. [41] to develop two DNAzymes, namely, IGF-IIP3-DRz1 and IGF-IIP3-DRz2, and to evaluate their antitumorigenic properties in various HCC cell lines. DRz1 was shown to be more effective than DRz2 in downregulating the expression of IGF-IIP3 at both mRNA and protein levels. Furthermore, DRz1 inhibited cell proliferation and induced late-stage apoptosis and necrosis in the SMMC-7721 cell population. DRz1-mediated inhibition of expression of IGF-IIP3 downregulated the expression of procaspase-3 and procaspase-9 and concomitantly increased caspase-3 and caspase-9 suggesting its caspase-dependent apoptosis through the intrinsic and mitochondrial pathway [41].

Inhibitors of apoptosis proteins (IAP) are a family of proteins that regulate apoptosis and a variety of biological processes such as immune response, cellular stress, translation, transcription, cell proliferation, differentiation, motility, and signal transduction [64]. Survivin is one of the eight members of the human IAP family known as an apoptosis inhibitor and mitosis regulator [65]. It is overexpressed in tumor cells and promotes tumor cell progression by dysregulating apoptosis and cell division and promoting chemoresistance and survival of cancer stem cells [66]. Survivin is considered a potential tumor antigen as its expression is tumor-specific. The blockage of survivin by means of both immunotherapeutic and molecular approaches is emerging as a promising strategy in treating various cancers, for instance, pancreatic and breast cancer. SD is an antisurvivin DNAzyme that was transfected into the human pancreatic carcinoma cell line (PANC-1) to examine its effectiveness as a tool in pancreatic cancer gene therapy [67]. It was observed that SD was capable of effective target cleavage, increasing apoptosis, and inhibiting the growth of PANC-1. It was suspected that SD induced apoptosis of PANC-1 through the activation of caspase-9 though it remains to be confirmed. Furthermore, SD could block the cells from entering into the S phase and inhibited PANC-1 cells from passing through the G2/M checkpoint resulting in inhibition of cell proliferation [67].

DRz1 and DRz2, another two survivin-specific DNAzymes, were transfected into MCF-7 to evaluate their potential as a breast cancer gene therapy. The DNAzymes were capable of downregulating the expression of survivin at both mRNA and protein levels. DRz1 exhibited a profound effect in inhibiting cell proliferation, inducing late-stage apoptosis, and suppressing motility and migration of MCF-7 cells. The downregulation of the expression of procaspase-3 and procaspase-9 and concomitant increase in caspase-3 and caspase-9 suggested the probable caspase-dependent apoptosis by DRz1 through the intrinsic and mitochondrial pathway [68].

The Bcl-2 family of proteins is well-known for their involvement in regulating apoptotic cell death [69]. Some members of the Bcl-2 family induce apoptosis (proapoptotic—Bax, Bcl-XS, Bak, Bad, Bik, Bid, Bim, Hrk, and Bok) while others suppress apoptosis (antiapoptotic—Bcl-2, Bcl-XL, Mcl-1, A1/Bfl-1, and Bcl-W) [70]. Antiapoptotic proteins such as Bcl-2 and Bcl-XL are frequently overexpressed in a broad range of human cancers, including glioblastoma, colorectal carcinoma, and breast cancer [69]. The targeted knockdown or silencing of the antiapoptotic Bcl-2 family can induce apoptosis which can be implemented as a novel cancer treatment strategy. Resultantly, Yu et al. [69] designed Bcl-XL-DT882 DNAzyme and transfected it into various cancer cell lines to determine its capability in downregulating Bcl-XL expression. DT882 reduced the Bcl-XL gene expression and enhanced the release of cytochrome C, thereby inducing apoptosis via the mitochondrial pathway in tested cells. In addition, the combined treatment of DT882 and Taxol sensitized all the cancer cells to Taxol treatment and resulted in profound apoptosis. DT882-treated Taxol-resistant cells showed a reduction in cell survival, indicating the DNAzyme-mediated downregulation of Bcl-XL expression in reversing the chemoresistant phenotype of cancer cells. Furthermore, the combined application of DT882 and Taxol markedly inhibited PC3 tumor growth *in vivo*, representing the potency of DT882 as a chemoadjuvant in cancer therapy [69].

### 3.4. DNAzymes Targeting Cancer-Related Kinases

Protein kinases catalyze protein phosphorylation which is an indispensable mechanism in regulating various cellular functions, including proliferation, motility, cell cycle, apoptosis, growth, and differentiation [71]. Deregulation of kinase activity can result in dramatic changes in these processes and are frequently found to be oncogenic, contributing to cancer cell survival and spread [72].

Aurora kinases are highly conserved serine/threonine kinases consisting of three members: Aurora-A, Aurora-B, and Aurora-C [73]. Among them, Aurora-A, located on the centrosome, is required for centrosome separation [74], bipolar-spindle assembly [75], and mitotic entry [76]. Its amplification and upregulation can induce chromosomal instability, leading to aneuploidy and cell transformation in several types of cancers [77], such as the bladder, breast, gastric, prostate, and colorectal cancer [73], making Aurora-A a promising target in cancer therapy.

The Aurora-A-DZ2 DNAzyme was evaluated for its efficacy against the progression of the PC-3 cell line both *in vitro* and *in vivo*. Upon transfection, DZ2 resulted in inhibitory effects on cell proliferation, cell cycle progression, and cell migration. Moreover, DZ2 markedly increased the caspase-3 and attenuated c-Myc and hTERT, reducing telomerase activity and ultimately enhancing apoptosis. Apart from the contribution of telomerase inhibition, induced apoptosis is also thought to be linked to other mechanisms that come to the surface through the DNAzyme-mediated Aurora-A knockdown [78]. These *in vitro* findings are compatible with Xing et al. [79] who used N-acetyl-L-leucine-polyethyleneimine (*N*-Ac-L-leu-PEI) as a carrier, thereby achieving an excellent delivery of the DNAzyme into PC-3 cells. Importantly, multipoint intratumoral injection of DZ2 to a human prostate cancer xenograft in nude mice produced a distinct tumor growth inhibition without noticeable adverse effects *in vivo*. These findings suggest the feasibility of DNAzymes targeting Aurora-A in the treatment of prostate cancer.

Philadelphia chromosome (Ph) is the hallmark of chronic myeloid leukemia (CML) [80] along with some other leukemias, including acute lymphoblastic leukemia (ALL), acute myeloid leukemia (AML), chronic neutrophilic leukemia (CNL), and mixed phenotype acute leukemia (MPAL) [81]. It is generated when the protooncogene Abelson murine leukemia gene (ABL) translocates to the breakpoint cluster gene (BCR), forming a BCR-ABL fusion gene [81].

According to different breakpoints in the BCR gene, three other BCR-ABL proteins are formed: p190 (e1:a2), p210 (b2:a2 and b3:a2), and p230, which are usually associated with ALL, CML, and CNL, respectively [82]. Both p190 and p210 have augmented tyrosine kinase enzymatic activity [10], resulting in continued cell proliferation, inhibited cell differentiation, and apoptosis [80]. Although tyrosine kinase inhibitors (TKI) belonging to different generations are utilized to treat CML [83], DNAzymes targeting BCR-ABL mRNA to inhibit gene expression and cell growth seem to be striking strategies when dealing with CML. S1bcrGUDz is one such DNAzyme targeting p210^BCR-ABL^ (b3:a2) mRNA. S1bcrGUDz was capable of specifically inhibiting the target protein expression and cell growth upon transfection into Ph^+^ cells [10]. When a Ph+ cell line (BV173) transfected with p210^BCR-ABL^ (b2:a2)-MeODz3 DNAzyme resulted in induced apoptosis without any off-target effects [84], the T315I mutant of BCR-ABL has been reported to show resistance to TKI, such as imatinib and dasatinib, used in CML patients [85]. Consequently, Kim et al. [86] designed BCR-ABL_T315I_-T315IDz DNAzyme and transfected it into imatinib-resistant BaF3/BCR-ABL_T315I_ to identify its potential in downregulating BCR-ABL expression and restoring drug sensitivity. Exposure to T315IDz effectively reduced the expression of BCR-ABL_T315I,_ inhibited cell viability, suppressed the cell growth rate, and induced apoptosis by upregulating caspase-3 and caspase-7 activity. Notably, the combined treatment of imatinib and T315IDz markedly increased the frequency of apoptosis, revealing the ability of T315IDz to restore the imatinib sensitivity. DNAzyme-mediated BCR-ABL_T315I_ knockdown and inhibitory effect of imatinib on the PI3k/AKT signaling pathway together enhanced the effectiveness of combined treatment against leukemia cells. Despite these promising *in vitro* analyses, thorough *in vivo* studies are compulsory for the treatment of CML.

Acute promyelocytic leukemia (APL), a distinct subtype of AML [87], is characterized by a balanced translocation t(15;17) that involves the retinoic acid receptor *α* (RAR*α*) gene on chromosome 17 and promyelocytic leukemia (PML) gene on chromosome 15, ultimately resulting in a PML/RAR*α* fusion gene [88]. PML/RAR*α* is critical in the development of APL [89]. Almost all de novo APL patients undergo disease remission under the treatment of all-trans-retinoic acid (ATRA), with chemotherapy eventually giving rise to clinical ATRA resistance [90]. Kabuli et al. [89] probed the feasibility of a molecular approach using DNAzymes, DZ1 and DZ3, targeting a PML/RAR*α* fusion transcript in search of promising therapeutic tools for APL. Transfection of DNAzymes into the APL cell line (NB4) resulted in suppressing PML/RAR*α* expression at mRNA and protein levels, inhibited cell viability, attenuated cell proliferation, and induced apoptosis. The combined treatment of DNAzymes and ATRA produced similar effects on NB4 cells but more intensely than the DNAzyme treatment alone.

Akt (protein kinase B, PKB) is activated in cells upon exposure to the diverse array of stimuli such as hormones, growth factors, and extracellular matrix components in a phosphoinositide 3-kinase- (PI3k-) dependent manner [91]. It comprises three isoforms known as Akt1, Akt2, and Akt 3 [92]. Upon activation, Akt regulates the function of many cellular proteins involved in metabolism, apoptosis, proliferation, survival, growth, and angiogenesis [93]. It has been found that the activation of Akt contributes to cancer initiation and maintenance, confers resistance to chemotherapy and radiation, and is a low prognostic factor for many cancers [91]. Aberrant activation through amplification of Akt genes or mutations in the components of the PI3K/Akt signal transduction pathway boots cell proliferation and survival, thereby contributing to cancer progression [94].

Yang et al. [95] synthesized five DNAzymes targeting Akt1 mRNA and transfected them into the NPC cell line (CNE-LMP1) to determine their effects on cell proliferation and apoptosis in NPC. Among them, Dz2 downregulated the expression of Akt1 at both mRNA and protein levels, inhibited cell proliferation, and induced apoptosis via Bcl-2 and Bax molecules in tested cells *in vitro*. Moreover, intratumoral administration of Dz2 to NPC xenograft growth in nude mice attenuated solid tumor development *in vivo*. DRz1, another anti-Akt1 DNAzyme tested against a human thyroid cancer cell line (SW597) *in vitro*, downregulated the Akt1 expression, inhibited cell proliferation and invasion, and induced apoptosis via the caspase-dependent manner. Further, the DNAzyme-mediated downregulation of VEGF and MMP-9 is thought to be associated with inhibited invasion and motility of SW597 cells [91].

Protein kinase C (PKC) is a family of critical signaling molecules in the VEGF pathway. It modulates the effects of extracellular stimuli such as growth factors, hormones, and drugs and promotes lipid hydrolysis [96]. Specifically, PKC*α* is upregulated in certain cancers such as bladder, endometrial, and breast cancers and is downregulated in others such as colorectal cancer and malignant renal cell carcinoma [97]. This indicates its intricate and highly tissue-specific functions, which render it a limited success as a drug target for cancer. The involvement of PKC*α* in the growth and progression of lung carcinomas and gliomas is well documented [98, 99]. In this respect, Sioud and Leirdal [100] investigated the efficacy of PKC*α*-DRz4 DNAzyme as a means of therapeutic strategy against human glioma (T98G). Exposure to DNAzyme attenuated the expression of PKC*α* at the protein level, inhibited cell proliferation, suppressed the Bcl-XL expression, and induced apoptosis of T98G cells. As per *in vivo* treatment, improved survivability of rats bearing intracranial tumor BT_4_C under single injection of DRz4 in combination with the continuous delivery of endostatin symbolizes an attractive therapeutic model against malignant gliomas [101].

### 3.5. DNAzymes Targeting Cancer-Related Specific Alleles

Protooncogenes present in normal cells function as growth factors, inducers of cellular signals, and nuclear transcription factors controlling cell differentiation and proliferation [102]. Modifications of these genes known as oncogenes are crucial in the appearance of cancer cells. Chromosomal translocation, point mutation, deletion, amplification, and insertion activation are considered genetic changes and contribute to oncogene generation via protooncogenes [103].

The Ras gene family encodes three homologous proteins, namely, *H*-, *N*-, and *K*-Ras, that occur exclusively at the inner plasma membrane. Ras proteins are small GTPases that function as regulators of cellular signal transduction controlling cytoskeletal integrity, cell proliferation, cell differentiation, cell adhesion, apoptosis, and cell migration [82]. Point mutations in the Ras gene, typically at codon 12, 13, or 61, are frequently found in human cancers [104]. *K*-Ras mutations prevail in the pancreatic, endodermal, colorectal, biliary tract, cervical, and lung cancers [82], whereas *N*-Ras and *H*-Ras mutations are typically encountered in melanoma and bladder cancer, respectively [105]. These point mutations make the Ras proteins impervious to GTP-induced hydrolysis of GTP to GDP and remain in the activated form [105]. Activating mutations in Ras induce constitutive signaling to downstream Ras effectors such as PI3k/Akt and Raf-MEK-ERK pathways [106]. Inhibition of Ras effector pathways has paved the way for the development of novel anticancer therapeutics. Consequently, *K-Ras*(G12V)-DZ-A DNAzyme was examined using a human colon adenocarcinoma cell line (SW480) which contained homozygous *K-Ras*(G12V)(GGT→GTT) mutant to determine its biological activity. DZ-A reduced K-Ras(G12V) expression at mRNA and protein levels in SW480 cells. Although *K-Ras*(G12V)-specific DNAzyme was unable to inhibit the proliferation of tested cells, the treatment of DZ-A reduced IC_50_ of doxorubicin for SW480 cells indicating its ability to sensitize tested cells to anticancer drugs. Also, the combined effect of DZ-A and radiation synergistically reduced the viability of SW480 cells [107].

Mutations in the epidermal growth factor receptor (EGFR) are implicated in NSCLC [108]. EGFR mutations cause uncontrolled cell proliferation and metastasis, leading to cancer development [109]. EGFR genes in NSCLC patients bear mutations in exons 18-20, including deletions of amino acids from 746 to 750 in exon 19 or point mutations such as T790M in exon 20 and L858R in exon 21 [110]. The exon 19 deletion and L858R point mutation result in uncontrolled proliferation of cancer cells by enhancing the kinase activity of EGFR [108]. A DNAzyme, Ex19delDZ, was developed to specifically cleave mutant EGFR mRNA to suppress lung cancer cell viability. DNAzyme treatment effectively suppressed the mutant EGFR expression in PC9/GR cells harboring E746~A750 deletion and T790M point mutation along with a concomitant attenuation of EGFR downstream signaling factor ERK1/2. Ex19delDZ reduced PC9/GR cell viability and was superior to EGFR siRNA for suppressing lung cancer cell proliferation due to the combination of nonspecific oligonucleotide and sequence-specific EGFR mRNA cleavage effects. The CpG sites of the Ex19delDZ upregulated the toll-like receptor 9 (TLR 9), rendering it an immunostimulatory effect, and subsequent activation of the p38 MAP kinase pathway and IL-6 secretion corresponding to induced apoptosis in EGFR-mutant lung cancer cells. The dual impact of CpG-Ex19delDZ, TLR9 activation, and EGFR downregulation brought about apoptosis and strong suppression of lung cancer cell proliferation, making the DNAzyme a potential agent in lung cancer therapeutics [108].

### 3.6. DNAzymes Targeting Oncogenic Viruses

Viral infections account for a substantial proportion of human cancers and stand as etiological agents for approximately 12% of all human cancers worldwide [111]. Six human viruses have been identified by the International Agency for Research on Cancer (IARC) as “carcinogenic to humans.” These include Epstein-Barr virus (EBV), hepatitis B virus (HBV), human papillomavirus (HPV) of several types, human T cell lymphotropic virus type 1 (HTLV-1), hepatitis C virus (HCV), and Kaposi's sarcoma-associated herpesvirus (KSHV) [112]. Some viral proteins are directly involved in the dysregulation of cellular processes leading to tumor progression, and therefore, targeting these oncoproteins delivers an excellent potential for virus-associated cancer therapies.

EBV is a gammaherpesvirus that infects more than 90% of the world's population and is linked to several human malignancies, including Burkitt's and Hodgkin's lymphomas, gastric carcinoma, and NPC [113]. Latent membrane protein 1 (LMP1) is a significant oncoprotein encoded by EBV and is associated with tumor necrosis factor receptor-associated factors (TRATs), tumor necrosis factor receptor-associated death domain protein (TRADD), and receptor-interacting protein (RIP) [113]. LMP1 activates several signal transduction pathways, including NF-*κ*B, the mitogen-activated protein kinases JNK and p38, the small GTPase Cdc42, and the JAK/STAT cascade [114], and causes various downstream pathological changes in cell proliferation, antiapoptosis, and metastasis [115]. The downregulation of LMP1 expression has provoked many scientists in search of therapeutics against EBV-associated human cancers. Consequently, Lu et al. [116] formulated 13 different DNAzymes against LMP1 mRNA and transfected them into B95-8 cells. Three DNAzymes, namely, Dz1, Dz7, and Dz10, downregulated the expression of LMP1 and inhibited B95-8 cell growth by arresting them at the G0/G1 checkpoint. Furthermore, the concomitant downregulation of the Bcl-2 gene expression indicated a close relationship between the LMP1 and Bcl-2 at a gene-specific level and their involvement in apoptosis. The DNAzymes induced the release of cytochrome C from mitochondria confirming the apoptosis. These findings encouraged Ke et al. [117] to evaluate the therapeutic effects of LMP1-targeted DNAzymes against NPC growth *in vivo*. Resultantly, DZ509 DNAzyme was designed and injected directly into the C666-1 xenograft mouse model. Intratumoral administration of DZ509 downregulated the LMP1 expression, concomitantly suppressing the tumor growth. These results indicated the therapeutic implications of DNAzymes targeting LMP1 in the treatment of NPC, albeit challenging.

Cervical cancer is stated as the fourth most common cancer in women. The link between genital HPV infections and cervical cancer was first demonstrated in the early 1980s by zur Hausen [118]. HPV-16 is a high-risk HPV strain and is considered the principal causal agent in cervical cancer [119]. It encodes two oncogenes, E6 and E7, which are associated with the initiation and progression of cervical cancer [120]. E6 and E7 expression promotes cell proliferation and the chance of malignancy by inactivating two tumor suppressor proteins, namely, p53 and retinoblastoma proteins [121]. In attempts of unveiling potential treatment options for cervical cancer, Reyes-Gutiérrez and Alvarez-Salas, [120] developed two DNAzymes against HPV-16 E6/E7 mRNA, out of which only one, Dz1023-434, produced an efficient cleavage against a *bona fide* antisense window at nt 410-445 within HPV-16 E6/E7 mRNA under the physiological range of [Mg^+2^] (1-5 mM). Optimized and chemically modified Dz1023-434 named Dz434-LNA resulted in a profound cleavage of full E6/E7 transcripts. A cervical tumor cell line (SiHA) transfected with Dz434-LNA presented a sharp reduction in E6/E7 mRNA levels, resulting in decreased proliferation and induced apoptosis, suggesting that DNAzymes are a probable cervical cancer treatment option.

### 3.7. DNAzymes Enhancing Cell Radiosensitivity

Radiosensitivity is the response of the tumor to irradiation which is measured by regression extent, response velocity, and response durability [122]. It depends on several factors that include the ability to repair the damage, hypoxia, cell cycle position, growth fraction, and the volume of the initial tumor [122]. Tumor cells are sensitive to ionizing radiation (IR), and therefore, radiotherapy has become an apparent cancer treatment option. Radiotherapy induces DNA damage and triggers the production of reactive oxygen species (ROS) in cancer cells [123]. Radiotherapy combined with immunotherapy and chemotherapy reduces tumor oxygen consumption and alters tumor immune response causing substantial clinical improvements in many types of cancer [123].

NPC that arises from the epithelial lining of the nasopharynx is a highly metastatic cancer with unique clinical and pathological characteristics. NPC is highly radiosensitive, and therefore, radiotherapy or radiotherapy in combination with chemotherapy is the main treatment strategies [124]. However, along with radiotherapy, radioresistance exists in many cases causing locoregional recurrence and distant metastasis after radiotherapy, especially in patients with tumors in advanced stages (stage III or IV) [125]. As a consequence, the DNAzyme-mediated downregulation of LMP1 expression is found to be effective in enhancing the radiosensitivity of EBV-related malignancies.

In their study, Lu et al. [126] used three DNAzymes, Dz1, Dz7, and Dz10, targeting LMP1 mRNA and transfected them into CNE1-LMP1 cells. The DNAzymes inhibited the LMP1 protein expression in tested cells leading to an inhibition of cellular signal transduction pathways which are abnormally activated by LMP1 such as AP1, NF-*κβ*, and JAK/STAT, suppressed cell proliferation by arresting cells at the S phase, and induced apoptosis via the mitochondrial pathway by increasing the activity of caspase-3, caspase-8, and caspase-9 concomitantly downregulating the expression of the antiapoptotic Bcl-2 gene. Moreover, the combined treatment of DNAzymes and IR increased the apoptotic rate and inhibited cell proliferation and colony-forming ability indicating DNAzyme-mediated downregulation of LMP1 expression in sensitizing CNE1-LMP1 cells to irradiation. Further, the effects of Dz1 on tumor growth were tested using CNE1-LMP1 carcinomas grown in nude mice *in vivo*. Subcutaneous injection of Dz1 combined with radiation treatment resulted in marked suppression of LMP1 expression, which led to a prominent tumor size reduction and enhanced the radiosensitivity of NPC cells.

Hypoxia-inducible factor-1 (HIF-1) is a heterodimeric transcriptional factor composed of HIF-*α* and HIF-*β* [127] and plays key roles in regulating tumor radiosensitivity [128]. HIF-1 is activated by hypoxia that modulates many genes involved in regulating critical processes such as metabolism, proliferation, apoptosis, and angiogenesis [129]. VEGF is one such activated gene performing vital roles in regulating tumor angiogenesis and progression [130]. It also protects endothelial cells from the cytotoxic effects of irradiation, leading to tumor radioresistance [131]. Overexpression of HIF-1 at the protein level in tumor cells corresponding to inadequate radiotherapy response has been identified in many types of cancers [129]. HIF-1 is a powerful radioreceptor, and thus, its blockade is essential in enhancing tumor radiosensitivity.

Yang et al. [132] exploited the EBV-LMP1-Dz1 DNAzyme to elucidate its potential to enhance radiosensitivity of NPC by suppressing HIF-1/VEGF activity. Dz1-mediated LMP1 knockdown presented an antiangiogenic activity in tested cells by reducing the VEGF expression and its secretion and inhibiting the three-dimensional tubule formation. Moreover, upon treatment with Dz1, the levels of phosphorylated JNKs, total c-jun, and phosphorylated c-jun were reduced, and the expression of HIF-1 and VEGF was also downregulated. Dz1 treatment sensitized CNE1-LMP1 cells to radiation treatment with a marked reduction in cell viability. Notably, the combined therapy of Dz1 and IR brought about a more substantial effect on cell viability than treatments of DNAzyme or IR alone. As per *in vivo* analysis, the combined treatment of Dz1 and IR produced a prominent tumor growth suppression and enhanced the radiosensitivity of CNE-LMP1 xenograft in athymic nude mice.

After a successful preclinical study, Cao et al. [124] further investigated the safety and tolerability of Dz1 in treating EBV-associated NPC in a randomized and double-blind clinical setting with 40 patients either receiving Dz1 or saline intratumorally in conjunction with radiotherapy. Dz1 treatment resulted in a high tumor regression rate and impacted the microvascular tumor permeability. Importantly, Dz1 treatment neither resulted in any cardiovascular, renal, and hepatic event nor increased radiation-induced toxicity. This put forward the potency of the DNAzyme-based therapeutic approach in enhancing the efficacy of radiotherapy for cancer treatment.

### 3.8. DNAzymes Targeting DNA Methyltransferase

Epigenetics is the study of heritable changes in gene expression that are not attributed to alterations in the primary DNA sequence. Epigenetic regulation of gene expression is governed by several mechanisms such as methylation of DNA, modifications of histones, and positioning of nucleosomes along with the DNA [133]. Among these, DNA methylation poses a great impact on cell physiology thus making it one of the widely discussed entities of epigenetic modifications in mammals. Alterations in DNA methylation interfere with transcriptional regulation leading to various diseases including cancer [134]. DNA methylation consists of covalent addition of a methyl group from S-adenosyl-L-methionine to 5-position of the cytosine moiety [135]. The reaction is catalyzed by a group of enzymes termed DNA methyltransferases (DNMTs). Such 5-methylcytosines are usually observed within CpG dinucleotides. DNMTs are extremely important epigenetic regulatory enzymes that take part in several biological activities such as preserving chromosome stability and genome integrity, embryo development, cell differentiation, and growth of organisms [136].

Five members of the DNMT family have been identified in mammals, and these include DNMT1, DNMT2, DNMT3a, DNMT3b, and DNMT3L. Nevertheless, only three, DNMT1, DNMT3a, and DNMT3b, participate in the production of cytosine methylation [136]. DNMT1 contributes to maintaining the methylation status after DNA synthesis. Its aberrations in terms of mutations, high expression, and low expression are frequently observed in various types of cancers [136] such as colorectal [137], pancreatic [138], and gastric cancer [139]. Owing to its active involvement in the development and progression of tumors, DNMT1 has become an excellent target in cancer therapy. For instance, reactivation of tumor suppressor genes (TSGs) has been silenced mainly due to the overexpression of DNMT1 using DNMT inhibitors (DNMTis) [135]. Decitabine (5-aza-2′-deoxycytidine) and azacitidine (5-azacytidine) are such DNMTis that have been tested in both preclinical and clinical settings [135]. Although effective, certain drawbacks such as substantial toxicity, poor bioavailability, and instability in physiological media trigger the development of novel and high-specific DNMT inhibitors [140].

On that account, Wang et al. [135] constructed a DNMT1-DT433 DNAzyme and validated it against T24 cells for its target-specific effect on DNA methylation and cell proliferation *in vitro*. Nuclear extracts from DT433-transfected T24 cells resulted in 50% reduction in methylation activity. This indicates the effective transection of DT433 into cells and its subsequent nucleic entry causing DNAzyme-dependent reduction in DNMT1 enzymatic activity. DT433 treatment inhibited the DNMT1 expression concomitantly elevating the p16 mRNA level in T24 cells thereby reactivating the tumor suppressor p16 gene. Reactivation of the p16 gene is evident from the subsequent inhibition of cell proliferation in cells treated with DT433. Interestingly, the effects incurred by DT433 on DNMT enzymatic activity, DNMT1 expression, and reactivation of the p16 gene were comparable to those of 5-Aza. This emphasizes that DT433 is as effective as such commercially available DNMTis. This preliminary study lays an alternative foundation to target DNMT for cancer treatment. This strategy was assisted with further preclinical data, and a targeted drug delivery system would be effective in the development of potential cancer treatment options.

## 4. A Glance into DNAzyme Delivery Systems

DNAzymes indeed are beneficial compared to other common nucleic acid-based therapeutic strategies yet influenced by factors such as serum stability and efficient delivery when it comes to *in vivo* applications. DNAzymes need to travel from the administration site, through the circulation and the tumor stroma, ultimately to their target cancer cells. Nucleic acids are potentially immunogenic and typically require a delivery tool to be utilized as therapeutics. An ideal delivery system is characterized by high transfection efficiency with a high degree of target cell specificity, low occurrence of toxicity and immunogenicity, biodegradability, stability, simple formulation, and versatility [141]. Tremendous efforts have been made to develop DNAzyme delivery systems that encircle all these characteristics to the best possible extent.  Table 6 summarizes the different types of delivery systems evaluated for efficient delivery of DNAzymes into cells and tissues, and the main outcomes of the respective studies are presented herein.

## 5. Conclusion

DNAzymes have been extensively assessed as effective anticancer agents following their discovery a decade and a half ago. Accompanied by the pioneering works of Breaker and Joyce in 1994, several structural modifications have been introduced to DNAzymes to enhance the stability and potency as an anticancer therapeutic agent. Many preclinical studies using *in vitro* assays and animal tumor models have revealed that DNAzymes may have clinical utility as an anticancer agent. These come with inherent pros and cons that incite further investigations in making DNAzymes a reality in the clinical arena. The therapeutic potential of DNAzymes lies in the design of special delivery systems that circumvent natural barriers to DNAzyme transport. Attempts at drug delivery systems (DDSs) such as conjugations of DNAzymes to cationic liposomes, cationic polypeptides, and nanotechnology have been employed to achieve efficient intracellular delivery of DNAzymes. DNAzymes have started to step forward from preclinical to clinical scenarios in light of these advances. Thus far, few clinical trials have validated the safety and tolerability of DNAzymes as therapeutic adjuvants. Although the development of DNAzymes as drugs is in its early stages of evolution, the emergence of economic and feasible DNAzyme-based cancer therapy is not far.

## Figures and Tables

**Table 1:** Advantages and disadvantages of different structural modifications used in DNAzymes.

Structural modification	Advantages	Disadvantages	Key points	References
3′-3′ inverted nucleotide at the 3′ end	Increase stability and enhance catalytic activity	Slower product release rate	Counteract the degradation by 3′-exonucleases	[15, 142–144]
Phosphorothioate linkages	Increase stability	Affect cleavage efficiency, toxicity, and immunologic responsiveness and produce sequence-independent effects	Substitution of oxygen atoms with sulfur atoms affects the DNAzyme structure in a molecularity-dependent manner Counteract the degradation by exonucleases	[142–144]
Locked nucleic acids	Increase affinity for complementary sequence, increase stability, solubility, easily automated synthesis, and straightforward cellular delivery	Influence catalytic activity and biological potency	Increase in stability due to efficient base stacking by adopting A-form geometry and oxymethylene bridge link between 2′ and 4′ carbon atoms of a furanose ring Charged backbone facilitating lucid cellular transfection A change in the charge distribution of the minor groove wall furnish solvation properties	[142, 143, 145]

**Table 2:** Respective sequences of DNAzymes.

DNAzyme	DNAzyme sequence
*β*1DE-OME	5′-CAAGGTGAGGGCTAGCTACAACGAAATAGAAG-3′ [28]
DE*β*1	5′-CAAGGTGAGGGCTAGCTACAACGAAATAGAAG-3′ [30]
Dz720	5′-GAGCATCCAGGCTAGCTACAACGAGGGTGCTGT-3′ [34]
MMP-9 DNAzyme	5′-AGGCGCCCAGGCTAGCTACA ACGACTCCGCGGC-3′ [37]
AM9D	5′-GTGGTGCCAGGCTAGC TACAACGATTGAGGTCG-3′ [38]
DRz1	5′-GATTCCCAGGCTAGCTACAAC GATGGTGTCT-3′ [45]
ED5	5′-CCGCTGCCAGGCTAGCTACAACGACCCGGACGTTI-3′ [46]
DzF	5′-GCGGGGACAGGCT AGCTACAACGACAGCTGCATTI-3′ [50]
DT18	5′-AGAGTGAGGCTAGCTACAACGAGGAGT-3′ [53]
VEGFR-2 DNAzyme	5′-TGCTCTCCAGGCTAGCTACAACGACCTGCACCT-3′ [55]
Dz13	5′-CGGGAGGAAGGCTAGCTACAACGAGAGGCGTTG-TI-3′ [58, 60–63]
SD	5′-CCTCGGCCA GGC TAG CTA CAA CGA CCGCTCCGG-3′ [67]
DRz1	5′-GCCTCGGTCCGCTCCG-3′ [68]
DT882	5′-TTTTTATAAGGCTAGCTACAACGAAGGGATGGG-3′ [69]
DZ2	5′-TTAACAGGGGCTAGCTAC AACGACCTGAAAT-3′ [78]
S1bcrGUDz	5′-AGGGCTTTTGAAGGCTAGCTACAACGATCTGCT-3′ [10]
MeODz3	5′-CTGAAGGGGGCTAGCGTACAACGATTCTTCCCT-3′ [84]
T315IDz	5′-CATGAACTCAACCGTAGCTACAACGAGATGATATAG-3′ [86]
DZ1	5′-CTCAATGGGGCTAGCTACAACGATGCCTCCC-3′ [89]
DZ3	5′-CTCAATGGGGCTAGGCTACAACGATGCCTCCC-3′ [89]
Dz2	5′-TGGTCCACAGGCTAGCTACAACGACCTGCGGCC-3′ [95]
DRz1	5′-GTCGTCCAGGCTAGCTACAACGAGGGGTACC-3′ [91]
DRz4	5′-GTCAGCCAGGCTAGCTACAACGAGGTCCCCC-3′ [100, 101]
DZ-A	5′-CTACGCCAAGGCTAGCTACAACGAAGCTCCAACT-3′ [107]
Ex19delDZ	5′-GCTTTCGGTGTGGCTAGCTACAACGAGTTTTGATAG-3′ [108]
Dz1	5′-GCAAAGGAAGGCTAGCTACAACGAAGAGGACAA-3′ [116, 126]
Dz7	5′-AGGGAGTCAGGCTAGCTACAACGACGTGGTGGT-3′ [116, 126]
Dz10	5′-CGTGTTCCAGGCTAGCTACAACGAGGTCAGGGT-3′ [116, 126]
Dz509	5′-CAAAGGAGAGGCTAGCTACAACGACAACCAATA-3′ [117]
Dz434-LNA	5′-TTCAGGAGGCTAGCTACAACGAACAGTGG-3′ [120]
DZ1	5′-GCAAAGGAAGGCTAGCTACAACGAAGAGGACAA-3′ [124, 132]
DT433	5′-GGTTGGTGA GGCTAGCTACAACGA GGTTGTGCT-3′ [135]

**Table 3:** *In vitro* applications of DNAzymes in cancer treatment.

Carcinoma cell line/s	mRNA target	DNAzyme	Modifications	Delivery system	Outcome	Reference
CX1.1, HT29, LOVO, LS180, and PC-3	*β*1 integrin	*β*1DE-OME	2′-O-Methyl modifications at both the 5′ and 3′ ends	LipofectAMINE^TM^ reagent (Giboo BRL®).	Inhibition of adhesion and invasion	[28]
PC-3 and HT29	*β*1 integrin	DE*β*1	2′-O-methyl modifications at both the 5′ and 3′ ends	LipofectAMINE^TM^ reagent	Inhibition of adhesion and invasion	[30]
Saos-2	uPAR	Dz720	Phosphorothioate modifications in the last three nucleotides at both ends	Lipofectamine 2000 (Invitrogen)	Inhibition of invasion and metastasis	[34]
A549	MMP-9	MMP-9 DNAzyme	Phosphorothioate modification at the first and last two phosphodiester linkages	Oligofectamine (Invitrogen)	Inhibition of cell proliferation, adhesion, migration, and invasion	[37]
MDA-MB-231	MMP-9	AM9D	—	Lipofectamine 2000 (Invitrogen)	Inhibition of invasion	[42]
SMMC-7721	IGF-II	DRz1	Inverted thymidine at the 3′ position	Lipofectamine 2000	Inhibition of invasion, motility, migration, and adhesion	[45]
PC-3, MDA-MB-231, SaOS-2, 143B, SJSA-1, G292, and SW872	c-jun	Dz13	Inverted thymidine at the 3′ position	FuGENE6 (Roche)	Induction of apoptosis	[60]
SMMC-7721, HepG2, and Huh7	IGF-IIP3	DRz1	Inverted thymidine at the 3′ position	Lipofectamine® 2000 (Invitrogen)	Inhibition of cell proliferation and induction of apoptosis	[41]
PANC-1	Survivin	SD	5′ phosphorothioate linkage and 3′ SPACER-C3 cap	Oligofectamine (Invitrogen)	Induction of apoptosis and inhibition of cell proliferation	[67]
MCF-7	Survivin	DRz1	—	Lipofectamine 2000 (Invitrogen)	Inhibition of cell proliferation and migration and induction of apoptosis	[68]
PC-3, T24, A549, MDA-MD-231, B9-58, and HCT116	Bcl-xL	DT882	1, 3, or 5 phosphorothioate modifications at both ends	Tetra meso (4-methylpyridyl) porphyrin (TMP)	Induction of apoptosis and chemosensitivity	[69]
PC-3	Aurora kinase A	DZ2	—	FuGENE 6 (Roche)	Suppression of cell growth, inhibition of cell cycle progression, induction of cell apoptosis, and attenuation of cell migration	[78]
K562	p210^BCR-ABL^ (b3:a2)	S1bcrGUDz	Phosphorothioate modifications in the first two bases at 5′ end and the last two bases at 3′ end	Liposome (GS2888)	Inhibition of cell growth	[10]
BV173	p210^BCR-ABL^ (b2:a2)	MeODz3	2′-O-Methyl modifications at both ends	Lipofectin	Induction of apoptosis	[88]
BaF3/ BCR-ABL_T315I_	BCR-ABL_T315I_	T315IDz	Phosphorothioate modifications in the first two bases at 5′ end and the last two bases at 3′ end	Cells were transfected by electroporation using the Neon Transfection System (Invitrogen)	Inhibition of cell viability, suppression of cell growth rate, induction of apoptosis, and chemosensitization	[86]
NB4	PML/RAR*α* mRNA	DZ1 and DZ3	—	Dioleoyl-3-trimethylammonium propane (DOTAP) liposome (Roche)	Inhibition of cell proliferation, reduction of cell viability, and induction of apoptosis	[89]
CNE1-LMP1	Akt1	Dz2	Phosphorothioate modification at the first and last two phosphodiester linkages	Oligofectamine (Invitrogen)	Inhibition of cell proliferation and induction of apoptosis	[99]
SW597	Akt1	DRz1	5′ phosphorothioate linkage and 3′ CPG-amine C7 cap	Lipofectamine 2000	Inhibition of cell proliferation, induction of apoptosis, and inhibition of invasion	[91]
T98G	PKC*α*	DRz4	Phosphorothioate modifications at the antisense arms and within the pyrimidine residues of the catalytic core	Dioleoyl-3-trimethylammonium propane (DOTAP) liposome	Inhibition of cell proliferation and induction of apoptosis	[100]
SW480	*K*-Ras	DZ-A	Phosphorothioate modifications in the last three nucleotides at the 3′ end	Lipofectin (Invitrogen)	Sensitization to chemo- and radiation therapies	[107]
PC9/GR	Mutant EGFR	Ex19delDZ	—	Lipofectamine 2000 (Invitrogen)	Reduction in cell viability, suppression of cell proliferation, and induction of apoptosis	[108]
B95-8	LMP1	Dz1, Dz7, and Dz10	Two phosphorothioate modifications on both arms	Tetra meso (4-methylpyridyl) porphyrin (TMP)	Inhibition of cell proliferation and induction of apoptosis	[116]
CNE1-LMP1	LMP1	Dz1, Dz7, and Dz10	Two phosphorothioate modifications on both arms	Tetra meso (4-methylpyridyl) porphyrin (TMP)	Inhibition of cell proliferation, induction of apoptosis, and radiosensitization	[126]
SiHa	HPV-16 E6/E7	Dz434-LNA	Locked nucleic acid (LNA) modifications	Lipofectin (Invitrogen)	Inhibition of cell proliferation and induction of apoptosis	[120]
T24	DNMT1	DT433	—	Lipofectamine	Inhibition of cell proliferation	[135]

**Table 4:** *In vivo* applications of DNAzymes in cancer treatment.

mRNA target	DNAzyme	Animal	Xenograft	Dose regime	Outcome	Reference
*β*1 integrin	*β*1DE	BALB/cA nude (nu-/-)-B6.Cg-*Foxn1*^nu^ mice	PC-3 and CX1.1	Intratumoral administration of 1.25 *μ*g of *β*1DE every second day after the tumor volume reached 150 mm^3^ for three weeks	Inhibition of solid tumor growth	[26]
*β*1 integrin	DE*β*1	BALB/cA nude (nu-/-)-B6.Cg-*Foxn1*^nu^ mice	PC-3 and HT29	Intratumoral administration of 1.25 *μ*g of DE*β*1 per tumor eight times every second day after the tumor volume reached 80-150 mm^3^	Inhibition of solid tumor growth	[30]
MMP-9	AM9D	MMTV-PyMT transgenic mice	Breast tumor	When tumors were at early palpable size, intratumoral administration of 10 or 25 *μ*g of AM9D once per week for four weeks	39.5% and 50% reduction in tumor size, respectively, 77% reduction in MMP-9 mRNA level	[38]
Egr-1	ED5	Athymic Balb/c nude mice	MCF-7	Intratumoral administration of 20 *μ*L of ED5 with 1 *μ*L of FuGENE6 twice a week	Inhibition of solid tumor growth	[46]
	DzF	Balb/c nude mice	MDA-MB-231	When the tumors were palpable, intratumoral administration of 10 *μ*g of DzF twice per week in an injectate volume of 10 *μ*L	Inhibition of solid tumor growth	[50]
VEGFR-1	DT18	Athymic nude mice	CNE1-LMP1	When the tumor volume reached 60-100 mm^3^, intratumoral administration of 100 *μ*g of DT18 with 3 *μ*L of Fugene6, twice a week	Suppression of tumor growth, changes in tumor vasculature and vessel permeability	[53]
VEGFR-2	VEGFR2 DNAzyme	Athymic nude mice	MDA-MB-435	When the tumor was visible, four intratumoral administrations consisting his-lys polymer with 2.9 *μ*g of DNAzyme	75% reduction in tumor growth, reduction in blood vessel density, cell death in tumor periphery	[59]
c-jun	Dz13	C57BL/J6 mice	B16F10	Commencement of the experiment, subcutaneous administration of 200 *μ*L of vehicle containing 750 *μ*g of Dz13 and 2.5 *μ*L of FuGENE6 twice per week	60% reduction in tumor growth, inhibition of tumor vascular density	[58]
		Balb/c nude mice	SaOS-2	When the tumors were palpable, intratibial administration of Dz13 at 0.8 *μ*M and caspase-2siRNA at 4 *μ*M in 50% Matrigel	Induction of caspase-2 expression	[60]
		Mice	SW872	Commencement of the experiment, intramuscular administration of Dz13+FuGENE6 at an oligonucleotide concentration of 0.4 *μ*M into the hind limb	Inhibition of tumor growth	[61]
		Severe-combined immunodeficient and C3H/Hen mice	T79	After 15-20 days of dermal implantation, intratumoral administration of 20 and 40 *μ*g of Dz13 with DOTAP and DOPE twice per week	Inhibition of tumor growth and suppression of neovascularization	[66]
Bcl-xL	DT882	Balb/c athymic nude mice	PC3	When tumors reached 100-200 mm^3^, a dose rate of 12.5 mg/kg/d of saline solution containing DT882 over 14 days via a ALZET osmotic pump (BioScientific)	Inhibition of tumor growth and chemosensitization	[69]
Aurora kinase A	DZ2	Balb/c nude mice	PC3	When the tumor reached about 65 mm^3^, intratumoral administration of 8 *μ*g of DZ2 daily for 14 days	Inhibition of tumor growth	[78]
Akt1	Dz2	Balb/c nude mice	CNE1-LMP1	When the tumor volume reached 60-100 mm^3^, intratumoral administration of 10 *μ*g of Dz2 with 3 *μ*L of FuGENE6 twice per week	Inhibition of tumor growth.	[95]
PKC*α*	DRz4	Inbred B.D.-IX rats	BT_4_C	Single intracranial administration of 100 *μ*g of DRz4 with 5 *μ*L of saline	Enhancement of survivability of tested animals	[101]
LMP1	DZ509	Balb/c nude mice	C666-1	When tumor size reached 5-8 mm, intratumoral administration of 33 *μ*g of DZ509 once per day for a week	Suppression of tumor growth	[117]
	Dz1	Athymic Balb/c nude mice	CNE1-LMP1	When the tumor volume reached 60-100 mm^3^, intratumoral administration of 20 *μ*L of Dz1 with 1 *μ*L of FuGENE6 twice a week	Suppression of tumor growth and radiosensitization	[126]
	Dz1	Athymic Balb/c nude mice	CNE1-LMP1	When the tumor volume reached 60-100 mm^3^, intratumoral administration of 100 *μ*g of Dz1 with 3 *μ*L of FuGENE6 once every three days	Suppression of tumor growth and radiosensitization	[132]

**Table 5:** Clinical trials of DNAzymes in cancer treatment.

mRNA target	DNAzyme	Patient type	Phase	Trial size	Transfection reagent	Dose regime	Reference
c-jun	Dz13	BCC	1	9	DOTAP/DOPE	Three dose groups (10, 30, and 100 *μ*g of Dz13) with three patients per group. Single intratumoral administration of 50 *μ*L of Dz13 over four weeks	[63]
EBV-LMP	DZ1	NPC	1	40	Saline	Administration into the tumor under the local anesthetization via an epical endoscope at a dose of 6 mg of DZ1 + 0.1 mL of saline per injection twice weekly over seven weeks	[124]

**Table 6:** Types of DNAzyme delivery systems and the outcomes.

Delivery systems	Outcomes	References
Biodegradable poly(D, L-lactide-co-glycoid) copolymer (PLGA) microspheres	Efficient and sustained delivery	[146]
Cyclodextrin-containing polycation (CDP)	A rapid and efficient intracellular uptake into various cell lines	[147]
Branched polyethylenimine	Higher biocompatibility and efficient transfection. Less cytotoxicity and promote gene dissociation	[148]
N-Acetyl-L-leucine-polyethylenimine (N-Ac-L-Leu-PEI)	Efficient cellular uptake and exhibited protective function against nucleases	[79]
Dendrimers	Low toxicity, higher water solubility, and increased stability against hydrolysis	[149]
Modified fourth-generation dendrimers termed G4 (MeI)	Efficient DNAzyme delivery without substantial toxicity	[149]
Colloidal gold nanoparticles	Less toxic and an efficient DNAzyme transfection	[150]
Liposomes	Efficient targeted delivery	[151–153]
Chitosan nanoparticles	Effective cellular uptake ability, remain stable at room temperature for a month and for seven days in serum without a substantial loss in activity	[154–156]
	Toxic side effects such as myelosuppression, alopecia, and hepatic toxicity can be associated	[157]
